# Phase 1 MAD results for the ABCA1‐agonist CS6253 developed for the indication of APOE4‐associated AD shows favorable safety and PK and suggest amyloid clearance

**DOI:** 10.1002/alz70861_108623

**Published:** 2025-12-23

**Authors:** Jan O. Johansson, Bengt Winblad, Henrik Zetterberg, Jeffrey L. Cummings, Hussein N Yassine

**Affiliations:** ^1^ Artery Therapeutics, Inc., San Ramon, CA USA; ^2^ Karolinska Institutet, Stockholm, Stockholm Sweden; ^3^ Department of Psychiatry and Neurochemistry, University of Gothenburg, Gothenburg Sweden; ^4^ Kirk Kerkorian School of Medicine, University of Nevada Las Vegas, Las Vegas, NV USA; ^5^ USC Keck School of Medicine, Los Angeles, CA USA

## Abstract

**Background:**

CS6253 is an ABCA1 agonist facilitating lipidation of apolipoprotein E (apoE) particles in the brain being developed for the indication of apolipoprotein E APOE4)‐associated dementia in Alzheimer’s disease (AD) patients. The Phase 1 Single‐Ascending‐Dose (SAD) showed safety and favorable pharmacokinetics (PK). Here we present the multiple ascending dose (MAD) results.

**Methods:**

In the Phase 1 MAD (*n* =16), CS6253 or placebo were administered every 72 hours three times to men and women 50 years and older, stratified for presence of at least one APOE4 gene. Cohorts of 8 comprised 6 active and 2 placebo subjects. Cohorts 1 and 2 received CS6253 7.5 and 10 mg/kg iv bolus injection (or placebo), respectively. Cerebrospinal (CSF) was collected before any dosing by lumbar puncture and by CSF catheter for 24 hours in conjunction with the 3rd/last dose. Subjects were assessed for adverse effects, PK, ECG, clinical chemistry, and biomarker effects. ApoE and amyloid were analyzed by NulisaSeq (Alamar) and HDL size by 1D‐gel electrophoresis (Boston Heart), at various time points to understand effect‐duration.

**Results:**

No serious adverse events (SAEs) were observed in the study and Treatment‐Emergent Adverse‐Effects, ECG characterization and safety chemistry did not differ between CS6253 and placebo subjects. PK showed a terminal half‐life of 16‐30 hours with no drug accumulation by repeat dosing.

In plasma CS6253 increased small HDL particles several‐fold over <8 hours after dosing, with an approximately 20% increase in plasma apoE over 72 hours. In addition, plasma Ab42/40 ratio increased 18% over 72 hours, mainly due to an Ab42 increase. CSF analysis is ongoing.

**Conclusion:**

The CS6253 Phase 1 MAD study showed excellent safety, tolerance and PK. Furthermore, consistent with previous findings in mice and monkey models CS6253 increased plasma small HDL and apoE, and showed signs of amyloid clearance. CS6253 may have utility in ABCA1‐related diseases, including APOE4 AD, by correcting cholesterol homeostasis.

